# Alterations in viscera histoarchitecture and organosomatic index as biomarkers of toxicity induced by Aba-Eku and Olusosun solid waste landfill leachates in *Rattus norvegicus*

**DOI:** 10.5620/eaht.2024022

**Published:** 2024-06-27

**Authors:** Chibuisi Gideon Alimba

**Affiliations:** 1 Cell Biology and Genetics, Department of Zoology, University of Ibadan, Nigeria

**Keywords:** body weight, solid waste leachates, metals and organic compounds, histopathological alterations, organosomatic indices, rats

## Abstract

Solid waste disposal generates leachate, a mixture of deleterious chemical, physical and microbial contaminants, which poses risk to human and wildlife health. Leachate toxicity on relative organ weight and histopathology of important viscera in mammalian body is scarce. Leachate induced toxic effects on organosomatic indices and histopathology of vital mammalian organs were investigated. Wister rats were orally exposed to 1 – 25 % of raw and simulated leachates from Aba-Eku and Olusosun landfills for 30 days. At post-exposure, organosomatic index and histoarchitectural assessment of major viscera (heart, spleen, thymus and lungs) were conducted. The physico-chemical and organic compositions of the leachates were analysed using standard protocol. The tested leachates decreased weekly and terminal body weights, and altered organosomatic index of examined viscera in rats. The histoarchitecture of the investigated viscera revealed pathologies that ranged from mild to severe degeneration, cellular infiltration, haemorrhage, congestion, necrosis, disorganization of tissues and vacuolations. Others include increased histiocytes within the bronchial associated lymphoid, lymphoid depletions, haemosiderin deposits and apoptosis were observed in the examined viscera. Physico-chemical analysis of the leachates showed different concentrations of toxic metals, PAHs and PCBs that were higher than national and international permissible limits allowed in wastewaters. The physico-chemical compositions of the leachates are capable of eliciting the observed alterations in organosomatic indices and histopathological lesions in mammalian viscera. Xenobiotic components of the leachates possibly generated free radicals and/or directly disrupted the organ architectures. These findings suggest health risk to wildlife and human population exposed to emissions from waste landfills.

## Introduction

Anthropogenic activities in industries, agriculture, medicine, municipality and educational institutions generate solid wastes, which are capable of releasing several thousands of chemicals into the environment [1, 2]. Disposal of solid wastes generated from anthropogenic activities into the environment is increasing around the globe at alarming rate. In some parts of the world, most importantly in developing countries, solid wastes are indiscriminately disposed into unsanitary landfills, combusted in open canals along major roads, and illegally disposed into sea and water ways and other parts of the environment [1, 3]. These awkward methods of disposing solid wastes have elicited strong national and international concerns due to contamination of the environmental metrices (air, soil, surface and underground water and biota of the ecosystem) and health risks associated with living within the vicinity of these wastes [1-4]. Landfilling is the most common method of managing solid wastes all over the world. It accounts for the management of about 95% of the total solid waste collection worldwide [5]. In Nigeria and most other developing nations, landfills are unsanitary; usually unlined and are located in public places surrounded by residential quarters and in wetland or other places with seasonally high-water tables [1, 2, 6]. This disposal method is capable of releasing chemicals in the forms of gases and or liquid solutions called leachates, into the environment and can endanger the survival of wildlife and humans [1, 2, 7]. Landfill gases and leachates are complex mixtures of hazardous and deleterious chemicals produced when solid wastes decompose in landfills through complex and highly variable biological and chemical processes. They are usually released in high volumes into nearby ground and surface water and surrounding land as leachates, and or dispersed as aerosol and particulate matters in dust and air [1, 2]. Human and wildlife may be exposed to toxic chemicals from landfills via inhalation of gases and particles during the dispersion of landfill gases or leachates into the air and or soil; ingestion of chemicals via consumption of leachate contaminated water and food, and dermal contact with these chemicals while working on landfills without adequate protection [1, 2]. Gaseous and leachate emissions from solid wastes pose serious threats to the environment and ecosystem due to chemical, radioactive elements and microbial composition of the emissions which contaminate the environment, and increase health risk among individuals residing and working within the vicinities of landfill sites [1, 2, 7, 8]. Chemicals in Aba-Eku and Olusosun landfill leachates reduced body weight and altered relative weight of liver and kidney in rats exposed for 30 days [9] and induced changes in haematological indices and erythrocyte morphology of exposed rats [10]. Furthermore, in a 30 day sub-chronic toxicity study using Wistar rats, Aba-Eku and Olusosun landfill leachates induced structural and functional alterations in brain of exposed Wistar rats, via alteration in the histology of the brain and antioxidant enzyme activities and lipid peroxidation status of the brain [11]. The reports of liver dysfunctions [12], brain damage [13], and incidence of low birth weight [14] recorded among human populations residing in proximity to landfills, were associated with exposure to hazardous chemicals emitted from the landfill. These reports may have increased the interest of scientists and epidemiologists to investigate possible adverse health effects associated with exposure to landfill emissions (landfill gases and leachates).

Living organisms are made up of different organ systems which are coordinated in a manner that bring about the normal functioning of the organisms. The systemic component is the highest level of biological organization in eukaryotes, and it comprises the organelles, cells, tissues and organs [15]. The systems in the body are involved in assimilation, metabolism, detoxification, storage, immunity, and elimination of xenobiotics and their metabolites from the body of organisms. They are vulnerable to direct pathological lesions and oxidative stress from the actions of xenobiotics, and are important target organs for xenobiotic induced injuries. Most of the organ systems are on the priority list of Agency for Toxic Substances and Disease Registry (ATSDR) as organs mostly affected by chemicals from landfills [16]. Xenobiotic induced alterations in the organ system of organisms can be reflected on the total functioning and coordination of the body. These alterations can be easily assessed from changes in the histopathology of the organs [17, 18]. Alterations in the organ systems induced by xenobiotics, are observed in the body and organ weight of the organisms [2]. It may also affect the general behaviour and functions of the organisms [18]. Alteration in body and organ weight is a sensitive biomarker that signals chemical induced damage to the body systems, and had been widely accepted as biomarker of assessment of chemicals associated with toxicities [18, 19]. When chemicals or xenobiotics induce toxicity in exposed organisms, the organto-body weight ratio of the body viscera, is altered when compared with the control [19]. The Society for Toxicologic Pathology (STP) recommended that organ weight be included in rodent toxicity experiment that last longer than 7 days [19]. Change in organ weight suggests alterations in cellular and tissue compositions of the organs including immune stimulation or depletion (sensitive indicator of immunotoxicity), stress and physiological perturbations. Histopathological evaluation of tissues is useful in identifying the type of lesions caused by xenobiotics and has been acknowledged the most sensitive end point for detecting organ toxicity [20]. It is also useful in providing information about acute or chronic effects of toxic substances that may not be detected by other biomarkers. Furthermore, it correlates well with alterations in organ weight [21]. Information is relatively limited on leachates induced alterations in histopathology and organ weight of some perceived inconspicuous but highly important systemic viscera in mammals as indices of systemic toxicity. Therefore, the aim of this study is to investigate the systemic toxicity of exposure to landfill leachates on spleen, thymus, heart and lungs (perceived inconspicuous viscera of the mammalian body, unlike kidney, brain, liver, ovaries and testes) of Rattus novergicus (Wistar rats). The specific objectives are to assess the histoarchitectural alterations in spleen, thymus, heart and lungs of Wister rats exposed to Aba-Eku and Olusosun landfills. Also, to determine the possible change in organ to body weight of the viscera in the exposed rats compared to the control.

## Materials and Methods

### Description of Aba-Eku and Olusosun landfills, the study sites

Aba Eku landfill, located in Ona Ara Local Government Area of Ibadan (longitude 3o 5^1^ East and latitude 7o 23^1^ North), Oyo State and Olusosun landfill Ojota, located in Oregun area of Kosofe Local Government Area (longitude 3o 24^1^E and latitude 6o 34^1^ N), Lagos State were the selected study sites. The two States are known for their high population growth with many light and heavy industries which make them more urbanize than other States in Southwestern, Nigeria. The landfills are co-disposal landfills receiving all categories of wastes, from domestic, industrial, agricultural, commercial, medicals and institutional wastes from the public and private sectors. The two landfills emit landfill gases and leachates which endanger human and wildlife health [1, 2].

### Leachate sample collection and simulation from decomposed wastes

Raw leachates were collected from 20 different leachate wells (shallow cavities on the landfill sites where leachates are collected) from Aba-Eku and Olusosun landfills, and were thoroughly mixed to provide a representative sample for each of the landfills. Leachate simulation from decomposed solid wastes was done by randomly collecting municipal soil samples from 10 different spots on the landfills and were mixed together to make a single representative sample for each site. The raw leachates and soil samples were transported to the laboratory. The soil samples were air-dried for a period of 2 weeks, after which the coarse particles were ground into fine particles using a pestle and mortar, and sifted through a 63-µ m (pore size) sieve to obtain homogeneous mixtures for each landfill. These mixtures were used to simulate (25 %) concentration of the leachate in accordance with ASTM [22]. 250 g of the soil samples from each landfill was weighed into 2 L flask, and 1,000 mL of distilled water (w/v) added to form soil solution. This solution was mechanically stirred at an hourly interval using glass rod for 24 hours at about 26.7 °C. After 24 h, the suspensions were allowed to settle for at least 1h and the supernatant was filtered with 2.5 µ m filters (Whatman® No. 42) to remove suspended particles. The obtained filtrates and the raw leachate samples were centrifuged (UNISCOPE Refrigerated Centrifuge machine, Model SM902B, Surgifield Medicals, England) at 10,000 revolution per minute (rpm) for 10 minutes at room temperature. The supernatants from the leachate samples were aliquoted into 1.5 L sterile sample bottles, their pH measured and were labelled as Olusosun Raw Leachate (ORL), Olusosun Simulated Leachate (OSL), Aba-Eku Raw Leachate (ARL) and Aba-Eku Simulated Leachate (ASL). The leachate samples were considered as the stock samples and stored at 4 °C until used.

### Physicochemical parameters and heavy metal analysis

Physical and chemical components of the leachates were analysed according to standard methods by American Public Health Association [23]. Nitrate, ammonia, chloride, phosphate, sulphate, total hardness, total alkalinity, biochemical oxygen demand (BOD), chemical oxygen demand (COD) and total solids (TS) were determined. Heavy metal concentration: Iron (Fe), lead (Pb), copper (Cu), manganese (Mn), arsenate (As), cadmium (Cd), chromium (Cr) and nickel (Ni), were determined in accordance with USEPA [24]. 100 mL of each leachate was digested by heating with concentrated HNO_3_. The resulting volume of about 2 - 3 mL of the leachate solution was made up to 10 mL with 0.1N HNO_3_. The concentrations of selected metals were determined in the resultant solution using PerkinElmer® A3100 atomic absorption spectrophotometer. Polycyclic aromatic hydrocarbons (PAHs), polycyclic brominated diphenyl ethers (PBDEs) and polycyclic chlorinated biphenyls (PCBs) in the simulated soil samples (ASL and OSL) were analysed using gas chromatography-mass spectroscopy (GC-MS) (Perkin Elmer, Clarus 680C GC and Perkin Elmer, Clarus 600C MS) [7].

### Animal exposure, measurement of organosomatic indices and histological analysis

Male and female Wistar albino rats (6 - 7 weeks old), which were produced from parents bred for several generations, were obtained from the animal house of the College of Medicine, University of Ibadan, Nigeria. They were transported to the animal house unit of the Department of Cell Biology and Genetics, until they were about 8 - 9 weeks old with mean ± SD weight of 80.37 ± 2.14 g. The *Rattus novergicus* had access to drinking water and were feed standard rodent chow (Ladokun feed Nigeria® ) *ad libitum*. Distilled water (0.0 %) was used as control. All animals were maintained in an apparently clean laboratory with conditions of 12 hours dark and light cycle, and temperature of 26.7 ± 4 °C for a minimum of 14 days to allow them acclimatize to the new environment before the commencement of the systemic toxicity experiments. Guide for care and use of laboratory animals published by the US National Institutes of Health (NIH Publication No. 85–23, revised in 1996) [25], as approved by the ethical committee, University of Lagos for the use of animals in experimental studies was carefully adhered during the study. Five (5) male and female *Rattus novergicus* / cage / sex were randomly assigned to a concentration each of the leachate samples. Each animal in a group was gavage with 0.5 mL of 1.0, 2.5, 5.0, 10.0 and 25.0 % (leachate/distilled water, v/v) leachate concentrations for 30 consecutive days [9, 11]. Similar treatments (exposure duration and volume of distilled water) were concurrently given to the negative group. Body weights of each animal in the treatment groups and control were measured at the beginning of exposure and once during the weekly exposure for the whole duration using Acculab® USA, Model-vic-303 electronic analytical weighing balance. The body weight of each rat in the group was then determined using Equation 1. At post exposure, both leachate exposed and control rats were intraperitoneally injected with anesthetic combination of 100 mg/kg Ketamine and 10 mg/kg of xylazine (Rompun® ) for 30 minutes. The euthanised rats were dissected and the heart, lungs, spleen and thymus excised from both exposed and control animals, and weighed to determine the absolute organ weight. The relative organ weight was then determined using Equation 2. The organs were then fixed in Bouin’s fluid fixative. After 48 hours of fixation, tissues were dehydrated by passing through different concentrations of ethyl alcohol - water (50, 70, 90 and 100 %), cleared in xylene, infiltrated with paraffin, and sequentially embedded in fresh paraffin wax blocks using rotary microtome. Tissue sections of 3 - 4 µ m thick were cut and routinely stained with Haematoxylin-Eosin (H&E), mounted in neutral DPX medium for morphological examination. Pathologist evaluated the slides for histopathological alterations at x 400 magnification using (Olympus, Tokyo) light microscope. Photomicrographs were produced using Sony digital camera (DSC-S730).

Absolute organ weight and relative organ weight analyses in toxicological research are important endpoint for the identification of potentially harmful effects of chemicals. Absolute organ weight is the direct measurement of the organ following exposure. Due to body weight of an animal influences the organ weight, measurement of relative organ weight, which is organ weight to body weight ratio (equation 2), gives the actual interpretation of the effect of the tested substance on the organ [18, 19]. However, both absolute and relative organ weight are reported in studies [18, 19].


(1) Body weight index = Final weight of rat − Initial weight of rat/Initial weight of rat

(2) Relative organ weight index = Absolute organ weight/Final weight of rat

### Statistical analysis

Data obtained from the organosomatic indices were presented as mean ± SD of the sample size and /or percentage (%) change from control. One Way Analysis of Variance (ANOVA) was used to determine the significant (p < 0.05) differences among the treated and control group. The treated groups were compared with negative control using Multiple Comparison Procedure of the Dunnett Post-hoc Test at p < 0.05 levels of significance. All statistical analyses were conducted with Graphpad prism 5.0® computer programs

## Results

### Physico-chemical characteristics, heavy metal and organic analyses of the leachates

The organic compounds analysed in OSL and ARL are presented in Tables 1 and 2 as discussed in Alimba et al., [4, 7]. The physico-chemical analysis revealed that ORL and ARL had strong foul-smelling odour compared to OSL and ASL. Also, ORL and ARL were both dark brown in colour, while OSL and ASL were brown and pale brown colour respectively (Table 3). The pH values of the analysed leachates were within the range of neutrality and slightly alkalinity of the pH scale, and the values are within the recommended permissible limits as standards for wastewater discharge into the environment in Nigeria [26]. The values of COD, BOD, total solid, hardness, alkalinity, chloride, phosphate, sulphate, nitrate, ammonia, Cr, Ni, Mn, Cd, Pb, Fe, As and Cu analysed in the leachates, are higher compared to standard permissible limits [26, 27]. The concentrations of these parameters in the raw samples (ORL and ARL) are higher than in the simulated samples (OSL and ASL) (Table 3).

### Body weight and terminal body weight gain of leachate treated rats

The terminal body weight of leachate treated rats at the end of 30 days exposure decreased significantly (p < 0.05) in a concentration dependent manner compared to the control in both ORL and ARL exposed rats. While terminal body weight of OSL and ASL treated rats decreased in a concentration independent manner. Expressing the terminal body weight of the corresponding control rats as 100 %, the percentage change in terminal body weight of male rats exposed to ORL (39.13 %) was higher than that of ARL (38.94 %) at the highest concentrations of the leachates. Also, at the 25 % of the leachates, female rats treated with ORL (36.17 %) had reduced terminal body weight than ARL (35.25 %) (Table 4). Alterations in weekly body weight of rats exposed to different concentrations of ORL and OSL showed concentration dependent decrease in body weight of exposed rats when compared to the control at the end of each week during the duration of exposure. While the decrease in body weight of rats exposed to OSL and ASL during the four weeks exposure durations present concentration independent compared with the control rats (data not shown).

### Absolute and relative thymus weight of leachate treated rats

The absolute thymus weight of male and female rats exposed to ORL and ARL was significantly (p < 0.05) different from the control while only 25 % (male) of ASL and 10 and 25 % (female) of OSL treated rats were significantly (p < 0.05) different from the corresponding control (Table 5). The relative thymus weight in leachate treated rats insignificantly (p > 0.05) decreased from corresponding control groups. Absolute and relative thymus weight are lower in female rat than male. Also, absolute and relative thymus weight in ORL and ARL treated rats were lower than OSL and ASL treated rats (Table 5).

### Absolute and relative spleen weight of leachate treated rats

There was significant (p < 0.05) increase in both the absolute and relative spleen weight of leachate treated rats. Absolute and relative spleen weight significantly (p < 0.05) increased at high treatment groups in ORL and ARL male and female rats compared with corresponding control groups (Table 6). Male rats showed higher alterations in absolute and relative spleen weight than female rats. Also, absolute and relative spleen weight gains were higher in ORL and ARL treated rats than OSL and ASL treated rats.

### Absolute and relative lung weight of leachate treated rats

Absolute and relative lung weight significantly (p>0.05) increase in both male and female rats exposed to the leachate samples. This increase was significant at high concentrations of the tested leachates in both male and female rats. Rats exposed to ORL and ARL had higher absolute and relative weight than OSL and ASL exposed rats. Leachate treated male rats had higher value than female (Table 7).

### Absolute and relative heart weight of leachate treated rats

There was insignificant (p>0.05) increase in both absolute and relative heart weight in rats exposed to various concentrations of ORL and ARL samples. Using Dunnett multiple analysis (p<0.05), higher concentrations of the leachate induce significant increase in heart weight compared to the control (Table 8). Absolute and relative heart weight were higher in rats treated with ORL and ARL than OSL and ASL exposed rats. Also, Absolute and relative heart weight were higher in males than female rats.

### Histopathological changes in the viscera of leachate treated rats

The histopathological lesions observed in the exposed rats were related in both raw and simulated leachates from Aba Eku and Olusosun municipal landfill. This may suggest similarity in the composition of the leachates. However, raw leachates induced more lesions on the tissues than the simulated leachates. Furthermore, there was no sex differences in the pattern or severity of induced pathological lesions by the tested leachates. Selected slides showing all possible lesions induced by tested leachates in the viscera (tissues) are used as representative figures in this study.

#### Histopathological changes in the thymus of treated rats

**Figure 1.**
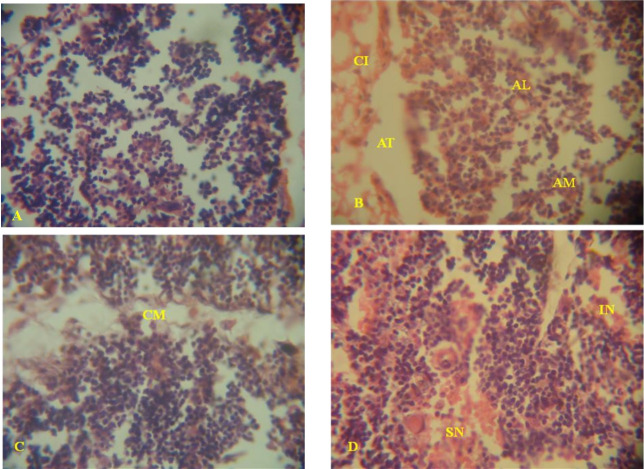
Histological section of the thymus from leachate treated rats and the control: (A) Histopathology of the cortical section of the thymus from the control rat showing normal distribution of lymphocytes and macrophages. (B) Thymic histology from ORL (10 %) treated rat showing severe depletion of the thymic lobules and increase in number of apoptotic lymphocytes (AL) and macrophages (AM). Also, there is cellular infiltration (CI) in the adipose tissues (AT). (C) Thymic histology from ORL (25 %) exposed rat showing indistinct cortico-medullary boundary (CM) with cellular infiltration. (D) Thymic histology from ARL (25 %) exposed rat showing severe necrosis (SN), infiltrations of macrophages (IN) and lymphoid depletion. H & E, x 400.

**Figure 2.**
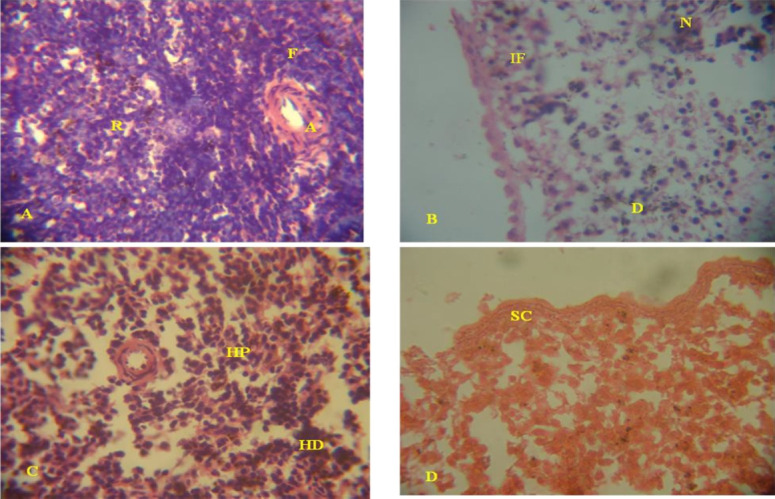
Histological section of the spleen from leachate treated rats and the control rat. (A). Histopathology of the spleen from the control rat showing normal follicle (F) around the arterioles (A) and normal red pulp (R). (B). Spleen histology from ORL (25 %) exposed rat showing marked lymphoid depletion characterized by degeneration (D), necrosis (N) and inflammatory (IF) change. (C). Spleen histology from ORL (10 %) exposed rat showing red pulp containing foci of haemosiderin ladened macrophages (HD) and hyperplasia of the periarterial (HP) splenic follicle. (D). Spleen histology from ARL (25 %) exposed rat showing severe necrosis and congestions of the sinuses and the cords. H&E, x 400.

The histology of the thymic cortex obtained from the control rat showed normal distribution of lymphocytes and macrophages without inflammatory cells (Figure 1A). Thymic histology of rats treated with ORL revealed severe depletion of the thymic lobules, cellular infiltration in the adipose tissues and increase in the number of apoptotic lymphocytes and macrophages (Figure 2B). Thymic histology of ARL treated rat revealed depletion, blurring of the cortico-medullary margins of the thymic lobules and reduction in the cellularity of the thymic medulla due to apoptosis of lymphocytes (Figure 1C). Severe necrosis and inflammation which caused lymphoid depletion of the cortical tissues were observed in the thymic histology of ORL treated rats (Figure 1D).

#### Histopathological changes in the spleen of leachate treated rats

Spleen histology from control rats showed apparently normal follicles around the arterioles and normal red pulp (Figure 2A). Lymphoid depletions especially of the periarteriolar lymphoid sheath (PALS), haemosiderin deposits, severe congestion of the sinuses and the cords, degeneration, necrosis and inflammation were observed in the spleen histology of rats treated with higher concentrations of the landfill leachates (Figure 2B-D), mostly with OSL and AEL. Similar lesions as observed in raw leachates were observed in OSL and ASL treated rats, but the lesions were not as severe as in the raw leachates. The lesions were also not sex specific.

#### Histopathological changes in the lungs of leachate treated rats

**Figure 3.**
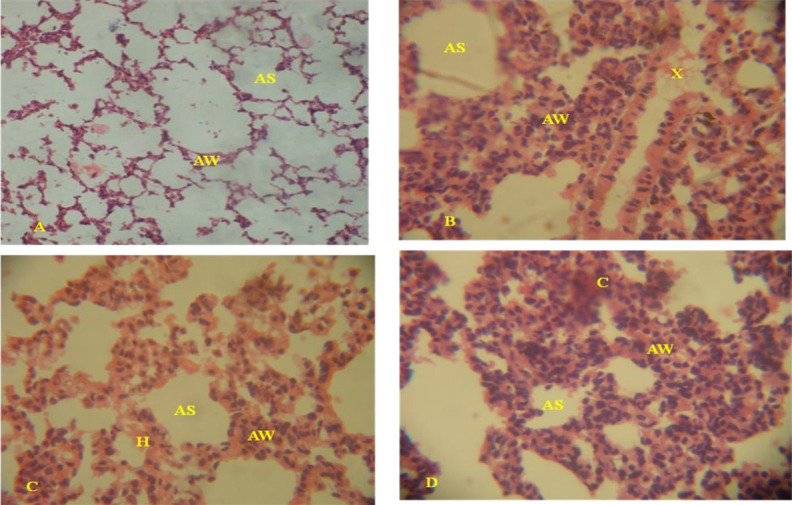
Histological sections of the lungs from leachate treated rats and the control: (A). Histopathology of the lung from the control rat showed normal alveoli with thin alveolar wall (AW) and normal alveolar sac (AS). (B). Lung histology from a ORL (25%) exposed rat showed moderate to severe wide spread proliferative thickening of the alveolar wall (AW). There are scattered foci of macrophages (X) with intracellular accumulation of black pigment (carbon particles). (C). Lung histology from ORL (10%) exposed rat showed localized severe thickening of the alveolar wall (AW). There are moderate increase histiocytes (H) within the bronchial associated lymphoid tissue (BALT) and moderate perivascular cuff. (D). Lung histology from ARL (25%) exposed rat showed moderate widespread proliferative thickening of the alveolar wall (AW). The pulmonary vessels are moderately congested (C) and there was lymphocytic perivascular cuff. H&E, x 400.

Histology of the lungs from the control rats showed normal alveoli with thin alveolar wall and sacs (Figure 3A). The histology of the lungs of rats treated with leachates showed spreads of proliferative thickened alveolar wall, scattered foci of macrophages with intracellular accumulated black pigmented carbon particles. Also, observed were localized severe thickening of the alveolar wall, increase histiocytes within the bronchial associated lymphoid tissue (BALT), presence of lymphocytic perivascular cuff and congestion of the pulmonary vessels (Figure 3B–D). Severity of the histopathological lesions for all tissues was observed in lungs of rats exposed to high leachate concentrations and observed lesions were not sex specific.

#### Histopathological changes in the heart of leachate treated rats

**Figure 4.**
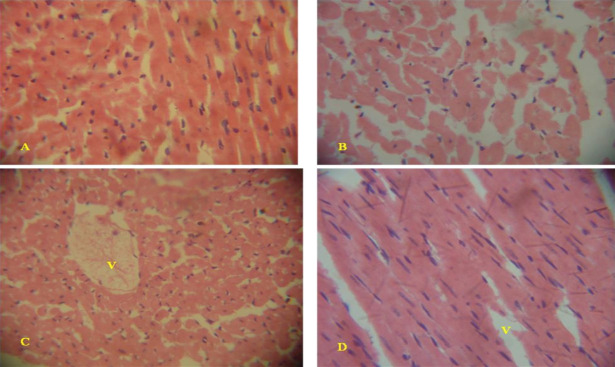
Histological sections of the heart from leachate treated rats and control: (A) Histopathology of the heart from the control rat with normal cardiomyocytes. (B) Histology of the heart from ORL (10 %) exposed rat showed normal architecture of the heart. (C) Histology of the heart ORL (25 %) exposed rat showed mild vacuolar degeneration of the cardiomyocytes (V). (D) Histology of the heart from OSL exposed rat showed slight extensions of vacuolar degeneration of the cardiomyocyte (V). H&E, x 400.

The histology of the heart from the negative control rat showed normal structure of cardiomyocytes with a nucleus in each of the myocytes (Figure 4A). Heart tissues from leachate treated rats showed mainly mild local and extended vacuolar degeneration of the cardiomyocytes in leachate treated rats (Figure 4B-D). The heart did not show severe lesions including necrosis, inflammations, congestion and haemorrhage of the cardiomyocytes unlike other examined viscera. Also, observed lesions were not sex specific.

## Discussion

Inappropriate disposal of municipal solid wastes by landfilling still poses concern to the public and environmental health in most countries of the world. The emissions (landfill gases and leachates) from decomposed wastes pollute the environment and endanger human and other biota of the ecosystem. Therefore, this study assessed alterations in the histology of the thymus, spleen, lungs and heart of rats exposed to landfill leachate and change in the organosomatic indices of these organs with the aim of understanding the possible mechanisms of landfill chemicals induced organ and or tissue damage. The simulated and raw leachates from both assessed landfills caused decrease in body weight of exposed rats (Table 4), and also caused decrease in thymic weight (Table 5), but increase the absolute and relative splenic (Table 6), lungs (Table 7) and heart (Table 8) weight in exposed rats compared to the control. The changes in organosomatic indices of the examined viscera, are in consonance with the observed alterations in the histology of the organs. Ranging from mild to severe histological lesions were observed in virtually all the examined viscera except the heart that was rarely affected. The observed lesions include cellular infiltrations (by neutrophils), increase in the number of apoptotic lymphocytes and macrophages (Kupffer cells), degenerations, congestions and necrosis in the thymus, spleen, and lungs, and mild vacuolar degeneration of the cardiomyocytes in the heart of the leachate treated rats compared to the control (Figures 1 - 4). Many of the toxic metals, physico-chemical constituents and deleterious organic compounds analysed in the leachates (Tables 1 - 3) were higher than the maximum permissible limits of these metals in wastewater from national [26] and international [27] regulatory authorities.

Alterations in absolute and relative weight of the evaluated organs are considered as sensitive indicators of immunotoxicities (immune stimulation or depletion), stress accumulation and physiological perturbation [29, 30]. It suggests that leachates from Aba-Eku and Olusosun landfills induced immune related toxicity on the spleen and thymus of exposed rats. The observation is supported by the findings of Rao et al. [30], that splenic weight increased and thymic weight decreased in albino rats exposed to 0.5 mL/kg body weight of kerosene for 6 days in a week for a period of 35 days. The authors concluded that the chemical composition of the kerosene interfered with immune system in the exposed albino rats. Also, that the tested leachates significantly increased absolute and relative lungs and heart weight may suggest that the compositions of the leachates are capable of eliciting pulmonary toxicity and cardiotoxicity in rats. Xinguo landfill leachate administered to mice altered kidney, liver, heart and spleen relative weights with concomitant increase in protein oxidation levels and DNA-protein crosslink formation in these organs compared to the control [31]. These findings implicated oxidative stress as culprit in organ weight alteration. It can be inferred that chemicals in the examined leachates are capable of altering the organosomatic indices of the body viscera of exposed rats. Findings from previous report showed that simulated and raw leachates from Olusosun and Aba-Eku decreased body weight and increased both absolute and relative liver and kidney weight in exposed rats [9]. Olusosun landfill leachate increased absolute and relative testes weight in mice [32], and increased absolute and relative brain weight in rats [11].

It can be posited that metals (Table 1) and organic compounds (Tables 2 and 3) present in the tested leachates, and acting individually or interacting as a mixture induced oxidative stress in the viscera of exposed rats. It is also possible that these chemicals directly altered the organosomatic indices of the selected viscera in the exposed rats. This assumption is corroborated by the findings that wild species of wood mouse, *Apodemus sylvaticus*, and greater white-toothed shrew, *Crocidura russula*, exposed to landfill emissions *in situ* from Garraf landfill, Spain, bioaccumulated toxic metals from the landfill with concomitant alterations in the weight of some selected organs [33]. In another study, Simmons et al. [34] exposed male F344 rats to 0.5 - 5.0 mL/kg of 10 different mixtures of organic and inorganic components obtained from wastes by oral gavaging for 24 h. The authors observed that nine among the mixture of assessed samples caused increase in relative liver weight and eight samples caused increase in serum alanine transaminase (ALT) and aspartate transaminase (AST) with concomitant induction of necrosis and degeneration in the centrilobular region of the liver. These findings further suggest oxidative stress induced damage to the body viscera as significant increase in transaminases indicate severe damage to organ tissue membrane [35].

The involvement of mammalian viscera in the modulation of biological processes including assimilation, metabolism, immunity, detoxification and elimination, increases the predisposition of these organs to direct pathological lesions and or oxidative stress induced damage associated with chemical constituents of the investigated leachates. Thymus is an exquisitely sensitive organ to stress and toxic insults. It is typically the first lymphoid tissue to respond to immunotoxicants during exposure to chemical substances [17]. The observed multiple histopathological changes in the thymus and spleen of leachate exposed rats could be attributed to the adverse immunotoxic effects of the xenobiotics present in the examined leachates [17, 36]. Ikechukwu et al. [37] exposed rats to different concentrations of crude oil for 28 days and observed degenerative, necrotic and inflammatory changes in the section of spleen from exposed rats. Histopathological lesions observed in the thymus and spleen of the leachate treated rats along with the concomitant changes in the organosomatic indices of these organs suggest that the activities of immune cells from these organs are compromised by chemicals present in the tested leachates. Histopathological changes in the lungs of leachate treated rats indicate that the lungs are susceptible to damage from chemical substances present in the examined leachates, irrespective of method of exposure, either by inhalation or oral gavaging of the leachates. Wildlife activities, on the landfills such as birds and rodents foraging for food remains from the solid wastes [2, 28], are exposed to leachates and landfill gases via drinking and inhalation, which may predispose the viscera including the lungs to pathological damage. Lungs have not been well assessed for histopathological lesions during landfill toxicity despite its frequent exposure to chemicals from landfill gases and leachates during incidental ingestion and inhalation. It is possible that the observed pathological lesions in the leachate treated rats were induced by oxidative stress or direct effects of chemicals in the leachates. Although, the histopathology of the heart in most of the leachate treated rats were apparently not severely affected as the thymus, spleen and lung, notwithstanding, the mild local vacuolar degeneration of the cardiomyocytes suggest serious morbidity since the heart is involve in pumping of blood for both pulmonary and systemic body circulation.

Chemicals in the leachates were possibly absorbed into the rat’s circulatory system and distributed during blood circulation to the body viscera. For instance, the lungs received blood containing chemicals from the leachate for oxygenation. These chemicals may injure the alveoli of the lungs. Following oxygenation, the blood is pumped from the heart to other parts of the body. During pumping action of the heart, the cardiomyocytes may be exposed to the chemical substances in the leachate. The histopathological lesions observed in the leachate exposed rats have been similarly reported in the liver biopsy from workers exposed to chemicals from industrial wastes [38] and in *Apodemus sylvaticus* and *Crocidura russula* (rodents) inhabiting Garraf landfill sites in Spain and exposed to emissions from the landfill in *in situ* [33]. The histopathological alterations in the examined tissues of the *Apodemus sylvaticus* and *Crocidura russula*, were linked to toxic metals; Pb, Hg, Cd, Fe, Mg, Zn, Cu, Mn, Mo and Cr, accumulated in the organs of the exposed rodents [33]. It is possible that haemorrhage, tissue degeneration, necrosis, cellular infiltration by macrophages and lymphocytes, inflammation depletion of tissues, and congestion of blood vessels observed in the examined viscera of leachate exposed rats were due to direct tissue damage by the toxic metals and organic compounds analysed in the tested leachates. Also, it is possible that these pathological lesions could have resulted from indirect tissue damage due to free radical species (reactive oxygen and nitrogen species) elicited by the landfill chemicals. This assertion lends credence to previous reports wherein Wistar rats were orally administered landfill leachates under laboratory-controlled conditions, and the pathological lesions observed herein in this study, were similarly reported. These include pathological lesions in the kidney and liver of Wistar rats exposed to Olusosun and Aba-Eku landfill simulated and raw leachates [9]; in pulmonary alveoli (lungs), renal cortex (kidneys), and spleen of rats exposed to leachates from open dump [39]; in brain tissues of rats exposed to Olusosun landfill leachates [11]; in testes of mice exposed to Olusosun landfill leachates [32]; and the heart, spleen, lungs, liver, kidneys and testes of free ranging black *Rattus rattus* caught from Abule Egba landfill [2]. It can be deduced from these *in vivo* and *in situ* studies that chemicals in landfill leachates can cause structural / pathological damage to most viscera of the mammalian body. Although, some viscera are at higher risk of being damage than others, when exposed to the toxic metals and other chemicals in leachates. This is due to their physiological functions, structural architecture, and locations in the body. For instance, the kidneys and liver are important target organs for xenobiotics due to their functions in metabolism, detoxification, storage, and excretion of chemicals [2, 9, 33, 39]. The testes and brain, due to their membrane-rich polyunsaturated fatty acids, low antioxidant status, and high iron contents, are more susceptible to lipid peroxidation and oxidative injury elicited by xenobiotics [2, 11], than the heart for instance. Also, due to the position and function of the lungs in gaseous exchange, inhaling air contaminated with landfill gases and particulate matters may increase risk to lung damage. Although, this study did not examine the detailed molecular mechanisms involved in the histopathological lesions induced by Aba-Eku and Olusosun landfill leachate exposed rats, however, previous report showed that Aba-Eku and Olusosun landfill leachates are capable of causing DNA damage and cytotoxicity in three human cell lines in in vitro via alterations in genes and processes involved in apoptosis and other forms cell death mechanisms [4, 7]. Another, important mechanism is alterations and total damage to the mitochondrial system of the cells [8].

Sexual dimorphism or gender differences, the state of being female or male, is increasingly considered in analytical research due to its influence in outcome of exposure to xenobiotics. Sex accounts for female-male toxicokinetic and toxicodynamic response to environmental chemicals and pharmaceuticals. Therefore, the consideration of sex in the assessment of xenobiotic induced toxicosis may define the effect of confounders in the outcome of research findings. Sex differences observed in the body weight, absolute and relative thymus, spleen, lung and heart weight of rats exposed to the tested landfill leachates may suggest differences in response to the chemical compositions of the tested landfill leachates. Although, the mechanisms involved is not known probably due to landfill leachate compositions are complex mixtures of various contaminants. However, differences in the expressions of sex hormones and certain drug and xenobiotic metabolising enzymes could alter the rate of absorption, distribution, metabolisms and elimination (toxicokinetics) of xenobiotics in the tested leachates, by the exposed male and female rats. Sex-related differences were not observed in the histopathological lesions induced in the various examined tissues. These observations are in consonance with previous studies wherein rats exposed to landfill leachates expressed sex related differences in body weight and absolute and relative liver and kidney weight, AST, ALT, albumin, total proteins, urea and creatinine but not in pathological lesions induced in the liver and kidney [9]. Furthermore, that Olusosun landfill leachates induced severe alterations in the examined biomarkers compared to Aba-Eku leachates, is related to the composition of wastes received in Olusosun landfill which is located in Lagos State, an industrialised State compared to Aba-Eku landfill which is situated in Oyo State, less industrialised [1, 2, 6]. Furthermore, the composition and concentrations of the heavy metals, physicochemical and organic composition of leachates from Olusosun landfill was higher that Aba Eku (Tables 1-3) [28, 32].

## Conclusions

Raw and simulated leachates obtained from Olusosun and Aba-Eku landfills altered organosomatic indices and induced histopathological dysfunctions in thymus, spleen, lungs and heart from exposed Wistar rats. The histological lesions observed in virtually all the examined viscera showed that there were severe cellular infiltrations (by neutrophils), increase in the number of apoptotic lymphocytes and macrophages (Kupffer cells), degenerations, congestions and necrosis in the thymus, spleen, and lungs but mild in the heart of leachate treated rats compared to the control. The inflammatory cells observed in landfill leachate treated rats were in response to tissue damage and their presence indicate endogenous generation of free radical species. This indicates that landfill leachate is capable of inducing significant deleterious alterations in the body viscera of wildlife and humans, if there is sufficient or continuous exposure to chemical substances from the landfills. The implication of these findings on exposed wildlife / human population may be grievous as the hazardous chemicals from the landfills can bioaccumulate and biomagnify along the food chain in the ecosystem. These findings are of great importance to nations that lack effective policy on solid waste management.

## Figures and Tables

**Table 1.** Identified organic compounds at their various peak retention times in Olusosun simulated soil leachate (OSL) sample [4], [7].

Peak retention time (min)	Identified organic compounds
2.03	Methylene chloride
2.64	[1,4]Dioxino[2,3-B]-1,4-hexahydro-2,3-dimethyl dioxin
6.32	1-[N-Dodecyl] Aziridine
6.81	N-methyl-N-Nitroso-1-octanamine
8.26	1-isopropoxy-2,2,3-trimethylaziridine(sin)
8.81	3,3'-Iminobispropylamine
9.66	1,2-Ethanediamine,N-(2-Aminoethyl)
10.91	1,3-bis-t-butylperoxy-phthalate
11.80	N-2-dimethyl-N-Nitroso-1-propanamine,
12.25	2-methyl-3-propyl oxaziridine,
12.97	4-Ethylamino-N-Butylamine
13.29	Azacyclodecan-5-ol
15.07	1,6-anhydro-2,4-dideoxy-beta-D-ribo-hexopyranose
16.02	2-trifluoroacetylamino propenoic acid
19.36	Cyanoacetylurea
20.32	Methanol, chloro-Acetate
20.97	Gamma-guanidinobutyric acid
21.38	Methyl iminodiacetic acid
21.56	2,2'-Iminobis[N-Hydroxyl ethane imidamide
21.84	2,3,4,5,6,7-hexahydro-3,6-dihexyl-10,11-diphenyl-bis[1,3]oxazino[6,5F]
22.03	2-Formyl-9-[Beta-D-Ribofuranosyl] Hypoxanthine
23.46	2-Decenoic acid
26.08	Pterin-6-carboxylic acid

**Table 2.** Identified organic compounds at their various peak retention times in Aba Eku simulated soil leachate (ASL) sample [4], [7].

Peak retention time (min)	Identified organic compounds
2.03	Methylene chloride
2.80	[1,4] Dioxino [2,3-B]-1,4-hexahydro-2,3-dimethyl dioxin
3.82	1-isopropoxy-2,2,3-trimethylaziridine(sin)
5.57	N-(2,2-dichloro-1-hydroxy-ethyl)-2,2-dimethyl-propionamide
6.17	1,6-anhydro-2,4-dideoxy-beta-D-ribo-hexopyranose
6.73	2-methyl-3-propyl oxaziridine
7.13	2-trifluoroacetylamino propenoic acid
8.94	4-Ethylamino-N-Butylamine
9.92	1-[N-Dodecyl] Aziridine
10.64	2,3,4,5,6,7-hexahydro-3,6-dihexyl-10,11-diphenyl-bis[1,3]oxazino[6,5F]
11.85	1,2-N-(2-Aminoethyl) ethanediamine
12.90	2,3-Epoxyhexanol
13.32	1,6-anhydro-3,4-dideoxy-beta-D-manno-hexa-pyranose
15.08	1,2-cyclopentanediol (trans)
15.37	Azacyclodecan-5-ol
15.61	1,3-Bis-T-Butylperoxy-phthalate
16.02	2-T-butylperoxy-2-ethylbutan-1-ol (Butyrate ester)
16.99	Gamma-guanidinobutyric acid
17.86	2,2,4-trimethyl-1,3-pentanediol
18.59	O-methyloxime decanol
18.98	1-Chloroacetyl piperidine,
19.56	2-Carbamyl-9-[Beta-D-Ribofuranosyl] Hypoxanthine
20.00	N-(2-Aminoethyl) 1,2-Ethanediamine
20.27	4-Biguanidoantipyrine
21.39	Ala-Gly,Trimethylsilyl Ester
22.47	Propanamide
23.96	2-Nonenoic acid
24.90	2-cyano Acetamide
25.71	1,3-bis-t-butylperoxy-phthalan
27.15	Emylcamate

**Table 3.** Physico-chemical parameters analyzed in Olusosun and Aba-Eku landfill leachates [9], [28].

Parameters^*^	ARL	ASL	ORL	OSL	NESREA^a^	USEPA^b^
Colour	Dark brown	Pale brown	Dark brown	Brown	-	-
pH	7.8	7.2	8.1	7.6	6.0 - 9.0	6.5 - 8.5
Nitrate	54.4	38.6	72.3	43.2	10	10
Ammonia	86.40	28.4	122.10	32.8	10	0.02
BOD^#^	601	397	594	306	50	-
COD^**^	512	410	487	398	90	-
Phosphate	122.02	98.86	215.70	106.13	2.0	-
Chloride	1106	1003	1,099	1012	250	250
Sulphate	114.34	109.35	218.12	122.51	250	250
Hardness	805	793	1,200	809	150	0 - 75
Alkalinity	502	439	623	456	-	20
TS^##^	3116.67	2300.50	4100.30	2200.60	-	-
Copper	2.44	0.92	3.86	1.02	0.5	1.3
Iron	3.20	1.87	4.71	2.03	-	0.3
Lead	2.08	0.81	2.00	1.18	0.05	0.015
Cadmium	1.44	0.31	2.20	0.65	0.2	0.05
Manganese	2.90	0.61	3.10	1.04	0.2	0.05
Arsenic	1.50	0.90	2.60	1.20	-	0.01
Nickel	1.88	1.96	2.51	2.12	0.05	-
Chromium	2.32	1.40	2.43	1.80	0.05	0.1

* All values are in mg/L except pH;

# BOD – Biochemical Oxygen Demand;

## TS – Total Solid;

** COD – Chemical Oxygen Demand;

a National Environmental Standards and Regulation Enforcement Agency (2011) (Nigeria) maximum permissible limits for wastewater [26].

b United State Environmental Protection Agency (2006) (www.epa.gov/safewater/mcl.html). [27].

- ARL – Aba-Eku raw leachates ; ASL – Aba-Eku simulated soil leachates ; ORL – Olusosun raw leachate; OSL – Olusosun simulated soil leachate.

**Table 4.** Percentage of the terminal body weight of rats exposed to ORL, ARL, OSL and ASL for 30 days compared to corresponding controls.

	Leachate Conc (%)	ORL (%)	ARL (%)	OSL (%)	ASL (%)
MALE	Dist. water	100	100	100	100
	1.0	66.65^b^	67.46^b^	89.08^a^	86.28^a^
	2.5	67.31^b^	67.67^b^	92.45	89.68^a^
	5.0	65.84^b^	68.58^b^	92.00	87.36^a^
	10.0	64.36^b^	66.45^b^	87.36^a^	86.32^a^
	25.0	61.87^b^	61.06^b^	85.63^a^	85.93^a^
FEMALE	Dist. water	100	100	100	100
	1.0	68.83^b^	70.02^b^	90.45^a^	92.24
	2.5	66.74^b^	70.78^b^	91.93	89.85^a^
	5.0	67.60^b^	67.49^b^	93.94^a^	92.79
	10.0	65.02^b^	66.68^b^	86.81^a^	85.89^a^
	25.0	63.83^b^	64.75^b^	88.03^a^	91.78^a^

ARL – Aba-Eku raw leachates; ASL – Aba-Eku simulated soil leachates; ORL – Olusosun raw leachate; OSL – Olusosun simulated soil leachate. Values are in percentages (%). Superscripts differ significantly from corresponding control value (a = p < 0.05; b = p < 0.01) by Dunnett’s multiple comparison posthoc test.

**Table 5.** Absolute and relative thymus weight of rats exposed to ORL, ARL, OSL and ASL for 30 days.

	Samples	ORL		ARL		OSL		ASL	
	Leachate Conc (%)	Abs. weight (g)	Rel weight (g)	Abs. weight (g)	Rel weight (g)	Abs. weight (g)	Rel weight (g)	Abs. weight (g)	Rel weight (g)
MALE	Dist. water	0.52 ± 0.01	0.27 ± 0.03	0.52 ± 0.01	0.27 ± 0.03	0.36 ± 0.03	0.21 ± 0.01	0.36 ± 0.03	0.21 ± 0.01
	1.0	0.33 ± 0.02^b^	0.25 ± 0.01	0.32 ± 0.05^b^	0.24 ± 0.04	0.31 ± 0.07	0.21 ± 0.06	0.31 ± 0.03	0.21 ± 0.07
	2.5	0.30 ± 0.06^b^	0.23 ± 0.03	0.32 ± 0.04^b^	0.24 ± 0.04	0.32 ± 0.03	0.20 ± 0.01	0.31 ± 0.06	0.21 ± 0.04
	5.0	0.29 ± 0.07^b^	0.22 ± 0.04	0.31 ± 0.07^b^	0.23 ± 0.04	0.31 ± 0.04	0.19 ± 0.01	0.31 ± 0.07	0.20 ± 0.21
	10.0	0.26 ± 0.06^c^	0.21 ± 0.06	0.29 ± 0.02^b^	0.22 ± 0.01	0.30 ± 0.04	0.20 ± 0.02	0.30 ± 0.05	0.21 ± 0.06
	25.0	0.20 ± 0.01^c^	0.16 ± 0.03	0.22 ± 0.02^b^	0.19 ± 0.01	0.26 ± 0.03	0.17 ± 0.03	0.28 ±.0.01^a^	0.19 ± 0.01
FEMALE	Dist. water	0.46 ± 0.02	0.25 ± 0.00	0.46 ± 0.06	0.25 ± 0.00	0.27 ± 0.04	0.17 ± 0.00	0.27 ± 0.04	0.17 ± 0.00
	1.0	0.22 ± 0.05^b^	0.17 ± 0.02	0.21 ± 0.06^b^	0.16 ± 0.02	0.22 ± 0.04	0.15 ± 0.01	0.23 ± 0.05	0.15 ± 0.01
	2.5	0.20 ± 0.01^c^	0.16 ± 0.03	0.21 ± 0.05^b^	0.16 ± 0.04	0.22 ± 0.07	0.15 ± 0.02	0.22 ± 0.07	0.15 ± 0.01
	5.0	0.20 ± 0.01^c^	0.16 ± 0.03	0.20 ± 0.02^b^	0.16 ± 0.03	0.21 ± 0.06	0.14 ± 0.01	0.20 ± 0.06	0.14 ± 0.04
	10.0	0.18 ± 0.04^c^	0.16 ± 0.03	0.19 ± 0.07^b^	0.15 ± 0.06	0.18 ± 0.09^a^	0.13 ± 0.05	0.20 ± 0.01	0.14 ± 0.04
	25.0	0.17 ± 0.00^c^	0.14 ± 0.00	0.17 ± 0.01^c^	0.14 ± 0.02	0.18 ± 0.01^a^	0.13 ± 0.04	0.18 ± 0.01	0.12 ± 0.01

Values are in means (± SEM). Superscripts differ significantly from corresponding control value (a = p < 0.05; b = p < 0.01; c = p < 0.001) by Dunnett’s multiple comparison posthoc test. Dist. water = distilled water (negative control). Abs. – Absolute weight, Rel – Relative weight; ARL – Aba-Eku raw leachates ; ASL – Aba-Eku simulated soil leachates; ORL – Olusosun raw leachate; OSL – Olusosun simulated soil leachate.

**Table 6.** Absolute and relative spleen weight of rats exposed to ORL, ARL, OSL and ASL for 30 days.

	Samples	ORL		ARL		OSL		ASL	
	Leachate Conc (%)	Abs. weight (g)	Rel weight (g)	Abs. weight (g)	Rel weight (g)	Abs. weight (g)	Rel weight (g)	Abs. weight (g)	Rel weight (g)
MALE	Dist. water	0.46 ± 0.04	0.23 ± 0.05	0.46 ± 0.04	0.23 ± 0.05	0.51 ± 0.05	0.29 ± 0.06	0.51 ± 0.05	0.29 ± 0.06
	1.0	0.51 ± 0.06	0.39 ± 0.04	0.50 ± 0.06	0.38 ± 0.02^b^	0.52 ± 0.05	0.34 ± 0.00	0.53 ± 0.05	0.35 ± 0.04
	2.5	0.52 ± 0.06	0.40 ± 0.01	0.44 ± 0.04	0.39 ± 0.05^b^	0.53 ± 0.03	0.33 ± 0.05	0.53 ± 0.02	0.35 ± 0.04
	5.0	0.61 ± 0.01^a^	0.47 ± 0.02^b^	0.58 ± 0.02	0.43 ± 0.02c	0.53 ± 0.10	0.33 ± 0.01	0.53 ± 0.06	0.35 ± 0.06
	10.0	0.67 ± 0.06^b^	0.48 ± 0.06^b^	0.61 ± 0.04	0.47 ± 0.03c	0.60 ± 0.06	0.40 ± 0.01	0.60 ± 0.01	0.40 ± 0.04
	25.0	0.77 ± 0.02c	0.63 ± 0.05c	0.73 ± 0.04c	0.61 ± 0.01c	0.61 ±.0.06	0.41 ± 0.04^a^	0.62 ±.0.05	0.41 ± 0.06
FEMALE	Dist. water	0.33 ± 0.05	0.18 ± 0.02	0.33 ± 0.05	0.18 ± 0.02	0.42 ± 0.05	0.26 ± 0.03	0.42 ± 0.05	0.26 ± 0.03
	1.0	0.37 ± 0.02^b^	0.29 ± 0.01	0.36 ± 0.03	0.28 ± 0.02	0.43 ± 0.05	0.26 ± 0.03	0.43 ± 0.05	0.29 ± 0.02
	2.5	0.50 ± 0.02^b^	0.40 ± 0.01^b^	0.43 ± 0.05	0.32 ± 0.02^a^	0.43 ± 0.02	0.30 ± 0.01	0.44 ± 0.02	0.30 ± 0.01
	5.0	0.52 ± 0.03^b^	0.41 ± 0.03^b^	0.51 ± 0.05^a^	0.41 ± 0.05^b^	0.45 ± 0.06	0.43 ± 0.01	0.44 ± 0.04	0.29 ± 0.01
	10.0	0.59 ± 0.05^c^	0.49 ± 0.02^c^	0.58 ± 0.02^b^	0.47 ± 0.03^c^	0.47 ± 0.02	0.34 ± 0.05	0.47 ± 0.09	0.34 ± 0.07
	25.0	0.60 ± 0.01^c^	0.51 ± 0.06^c^	0.59 ± 0.00^c^	0.49 ± 0.02^c^	0.57 ± 0.06	0.40 ± 0.01^b^	0.55 ± 0.04	0.37 ± 0.03

Values are in means (± SEM). Superscripts differ significantly from corresponding control value (a = p < 0.05; b = p < 0.01; c = p < 0.001) by Dunnett’s multiple comparison posthoc test. Dist. water = distilled water (negative control). Abs. – Absolute weight, Rel – Relative weight; ARL – Aba-Eku raw leachates; ASL – Aba-Eku simulated soil leachates; ORL – Olusosun raw leachate; OSL – Olusosun simulated soil leachate.

**Table 7.** Absolute and relative lung weight of rats exposed to ORL, ARL, OSL and ASL for 30 days.

	Samples	ORL		ARL		OSL		ASL	
	Leachate Conc (%)	Abs. weight (g)	Rel weight (g)	Abs. weight (g)	Rel weight (g)	Abs. weight (g)	Rel weight (g)	Abs. weight (g)	Rel weight (g)
MALE	Dist. water	0.90 ± 0.01	0.46 ± 0.05	0.90 ± 0.01	0.456 ± 0.05	1.21 ± 0.06	0.69 ± 0.02	1.21 ± 0.06	0.69 ± 0.02
	1.0	0.92 ± 0.01	0.70 ± 0.06^a^	0.92 ± 0.04	0.693 ± 0.01	1.22 ± 0.11	0.78 ± 0.05	1.22 ± 0.05	0.81 ± 0.05
	2.5	0.95 ± 0.03	0.75 ± 0.09^b^	0.94 ± 0.02	0.703 ± 0.04	1.22 ± 0.05	0.75 ± 0.04	1.22 ± 0.08	0.80 ± 0.06
	5.0	0.99 ± 0.15	0.77 ± 0.04^b^	0.99 ± 0.01	0.731 ± 0.06^a^	1.22 ± 0.04	0.76 ± 0.04	1.21 ± 0.05	0.79 ± 0.06
	10.0	1.19 ± 0.07	0.94 ± 0.02^c^	1.10 ± 0.05	0.871 ± 0.04^b^	1.26 ± 0.04	0.83 ± 0.05	1.25 ± 0.03	0.83 ± 0.04
	25.0	1.40 ± 0.12^b^	1.15 ± 0.05^c^	1.39 ± 0.02^c^	1.153 ± 0.05^c^	1.31 ±.0.13	0.88 ± 0.06	1.32 ±.0.02	0.88 ± 0.05
FEMALE	Dist. water	0.73 ± 0.03	0.39 ± 0.01	0.73 ± 0.03	0.39 ± 0.01	1.12 ± 0.12	0.69 ± 0.08	1.12 ± 0.12	0.69 ± 0.08
	1.0	0.88 ± 0.05	0.69 ± 0.02^b^	0.90 ± 0.06	0.67 ± 0.01	1.12 ± 0.11	0.76 ± 0.02	1.12 ± 0.04	0.75 ± 0.04
	2.5	0.96 ± 0.04	0.77 ± 0.01^c^	0.94 ± 0.07	0.71 ± 0.09	1.12 ± 0.06	0.75 ± 0.04	1.13 ± 0.11	0.77 ± 0.01
	5.0	0.97 ± 0.04	0.77 ± 0.01^c^	1.03 ± 0.10^a^	0.79 ± 0.06	1.13 ± 0.05	0.74 ± 0.04	1.13 ± 0.01	0.75 ± 0.04
	10.0	0.99 ± 0.07	0.82 ± 0.05^c^	0.99 ± 0.05	0.80 ± 0.00^a^	1.21 ± 0.09	0.86 ± 0.06	1.19 ± 0.03	0.86 ± 0.04
	25.0	1.22 ± 0.08^b^	1.02 ± 0.10^c^	1.20 ± 0.02^b^	1.00 ± 0.02^b^	1.24 ± 0.10	0.87 ± 0.04	1.26 ± 0.07	0.85 ± 0.04

Values are in means (± SEM). Superscripts differ significantly from corresponding control value (a = p < 0.05; b = p < 0.01; c = p < 0.001) by Dunnett’s multiple comparison posthoc test. Dist. water = distilled water (negative control). Abs. – Absolute weight, Rel – Relative weight; ARL – Aba-Eku raw leachates ; ASL – Aba-Eku simulated soil leachates; ORL – Olusosun raw leachate; OSL – Olusosun simulated soil leachate.

**Table 8.** Absolute and relative heart weight of rats exposed to ORL, ARL, OSL and ASL for 30 days.

	Samples	ORL		ARL		OSL		ASL	
	Leachate Conc (%)	Abs. weight (g)	Rel weight (g)	Abs. weight (g)	Rel weight (g)	Abs. weight (g)	Rel weight (g)	Abs. weight (g)	Rel weight (g)
MALE	Dist. water	0.43 ± 0.05	0.23 ± 0.10	0.43 ± 0.05	0.23 ± 0.10	0.92 ± 0.04	0.53 ± 0.04	0.921 ± 0.039	0.53 ± 0.04
	1.0	0.49 ± 0.04	0.37 ± 0.03	0.48 ± 0.02	0.36 ± 0.02	0.91 ± 0.03	0.59 ± 0.01	0.920 ± 0.029	0.61 ± 0.06
	2.5	0.51 ± 0.05	0.39 ± 0.04	0.49 ± 0.00	0.37 ± 0.02	0.90 ± 0.05	0.56 ± 0.03	0.911 ± 0.007	0.60 ± 0.06
	5.0	0.54 ± 0.01	0.41 ± 0.01	0.54 ± 0.10^a^	0.40 ± 0.06	0.92 ± 0.04	0.57 ± 0.03	0.903 ± 0.040	0.59 ± 0.02
	10.0	0.69 ± 0.01^c^	0.54 ± 0.05^c^	0.62 ± 0.02^c^	0.47 ± 0.05^a^	0.93 ± 0.09	0.61 ± 0.06	0.941 ± 0.024	0.62 ± 0.03
	25.0	0.73 ± 0.02^c^	0.60 ± 0.06^c^	0.7 ± 0.01^c^	0.58 ± 0.03^b^	0.95 ± 0.06	0.63 ± 0.05	0.944 ± 0.018	0.63 ± 0.03
FEMALE	Dist. water	0.36 ± 0.03	0.14 ± 0.09	0.361 ± 0.029	0.14 ± 0.09	0.59 ± 0.04	0.36 ± 0.03	0.59 ± 0.04	0.36 ± 0.03
	1.0	0.45 ± 0.04	0.35 ± 0.04^c^	0.429 ± 0.038	0.33 ± 0.06^a^	0.60 ± 0.02	0.41 ± 0.05	0.55 ± 0.05	0.39 ± 0.01
	2.5	0.45 ± 0.03	0.36 ± 0.02^c^	0.462 ± 0.008	0.35 ± 0.03^b^	0.60 ± 0.09	0.40 ± 0.05	0.54 ± 0.01	0.37 ± 0.02
	5.0	0.46 ± 0.03	0.37 ± 0.02^c^	0.463 ± 0.028	0.37 ± 0.024^b^	0.60 ± 0.04	0.39 ± 0.07	0.60 ± 0.01	0.40 ± 0.04
	10.0	0.49 ± 0.00	0.40 ± 0.02^c^	0.479 ± 0.047	0.39 ± 0.03^b^	0.69 ± 0.04	0.49 ± 0.04	0.63 ± 0.04	0.45 ± 0.04
	25.0	0.54 ± 0.02^b^	0.46 ± 0.01^c^	0.526 ± 0.053^a^	0.44 ± 0.03^c^	0.84 ± 0.03^c^	0.59 ± 0.04^b^	0.72 ± 0.04^a^	0.48 ± 0.00^a^

Values are in means (± SEM). Superscripts differ significantly from corresponding control value (a = p < 0.05; b = p < 0.01; c = p < 0.001) by Dunnett’s multiple comparison posthoc test. Dist. water = distilled water (negative control).
